# Impact of preconceptional micronutrient supplementation on maternal mental health during pregnancy and postpartum: results from a randomized controlled trial in Vietnam

**DOI:** 10.1186/s12905-017-0401-3

**Published:** 2017-06-17

**Authors:** Phuong H. Nguyen, Ann M. DiGirolamo, Ines Gonzalez-Casanova, Hoa Pham, Wei Hao, Hieu Nguyen, Truong V. Truong, Son Nguyen, Kimberly B. Harding, Gregory A. Reinhart, Reynaldo Martorell, Usha Ramakrishnan

**Affiliations:** 1 International Food Policy Research Institute, Washington, DC USA; 2 Thai Nguyen University of Pharmacy and Medicine, Thai Nguyen, Vietnam; 3 Georgia State University, Atlanta, GA USA; 4 Emory University, Atlanta, GA USA; 5 Micronutrient Initiative, Ottawa, ON Canada; 6 The Mathile Institute for the Advancement of Human Nutrition, Dayton, OH USA

**Keywords:** Preconception, Multiple micronutrient, Supplement, Women of reproductive age, Mental health, Randomized controlled trial, Vietnam

## Abstract

**Background:**

Micronutrient malnutrition has been associated with maternal depressive symptoms (MDS), but little is known about the effects of preconceptional micronutrient supplementation. This paper examined the effects of preconceptional micronutrient supplementation on MDS during pregnancy and postpartum.

**Methods:**

We used data from a double-blind controlled trial (PRECONCEPT) in which 5011 Vietnamese women were randomized to receive weekly supplements containing either a) multiple micronutrients (MM) b) iron and folic acid (IFA) or c) folic acid (FA) until conception (*n* = 1813). Maternal mental health was assessed using the Center for Epidemiologic Studies Depression Scale (CES-D) at baseline (preconception), and the Edinburgh Postnatal Depression Scale (EPDS) during pregnancy and 3 months postpartum. Elevated MDS was defined as EPDS score ≥ 4. All group comparisons were done using ANOVA or chi-square tests of proportions intention to treat and per protocol analyses (women consumed supplements ≥26 weeks before conception). We also conducted stratified analyses by preconception CES-D scores, underweight, or anemia status using generalized linear models.

**Results:**

Baseline CES-D scores were similar across treatment groups. The proportion of women experiencing elevated MDS was 11.3, 8.1 and 4.9% at first, second and third trimesters of pregnancy, respectively, and 3.6% at 3 mo postpartum. Mean EPDS scores at first (1.5 ± 2.7), second (1.1 ± 2.4), and third trimester of pregnancy (0.7 ± 2.0) and early postpartum (0.6 ± 1.8) were low and did not differ by treatment group. However, among women in the highest tertile of CES-D scores at preconception, mean EPDS scores in the first and second trimesters of pregnancy were lower in the MM and IFA groups compared to FA only (*P < 0.05)*.

**Conclusions:**

Weekly preconceptional micronutrient supplements containing iron did not improve depression measures relative to folic acid alone among all women, but may have benefitted women who were at risk for depression.

**Trial registration:**

The trial was registered retrospectively at ClinicalTrials.Gov as NCT01665378 on August 13, 2012.

**Electronic supplementary material:**

The online version of this article (doi:10.1186/s12905-017-0401-3) contains supplementary material, which is available to authorized users.

## Background

Strong evidence supports the importance of maternal mental health for optimal child health, nutrition and development [1–3]. Children exposed to maternal depression during the first 1000 days of life may experience irreversible changes in their brain architecture and/or persistent disruptions of their stress response systems, resulting in increased risk for long-term physical and psychological difficulties [4]. During pregnancy, depression may affect child outcomes (including behavior, socioemotional adjustment, and emotion regulation) through altered placental function, epigenetic changes in the child, and stress reactivity. During the postnatal period, maternal depression may affect child outcomes through altered mother–child interactions, environmental influences, and social support [5]. Poor maternal mental health, in particular maternal depression, is also a risk factor for higher morbidity and growth faltering in young children [6].

Research also suggests a relationship between nutrition and mental health status [7, 8]. Several micronutrients such as iron, zinc, folate, vitamin B-6, B-12, calcium, selenium, and n-3 fatty acids have been linked to maternal mental health and wellbeing [9, 10] However, much of the work to date has focused on the postpartum period. For example, higher depressive symptoms have been reported for women who were anemic during the postpartum period compared with non-anemic women [11]. Recent evidence from a randomized, placebo-controlled trial conducted during postpartum in South Africa demonstrated the benefits of daily iron supplementation in reducing depressive symptoms among anemic mothers [12]. In contrast, little is known about the benefits of providing micronutrient supplements before and during pregnancy for improving maternal mental health outcomes, indicating the need for studies that evaluate the effects of nutrition interventions during these critical periods. This is particularly relevant in low-middle income countries where the problems of maternal depression and poor health and nutrition among women and children are high and coexist [13–15].

We had the unique opportunity to include measures of maternal mental health as a secondary outcome in the PRECONCEPT study, a large randomized controlled trial (RCT) evaluating the effects of preconceptional micronutrient interventions on birth outcomes in rural Vietnam [16]. The objective of this paper was to examine the effects of weekly micronutrient supplements containing multiple micronutrients (MM), iron and folic acid (IFA) or only folic acid (FA), when provided to women of reproductive age before conception, on maternal mental health outcomes during pregnancy and postpartum in rural Vietnam.

## Methods

### Study context, design and participants

The study design, trial profile, and characteristics of the study sample have been described in detail elsewhere [16]. Briefly, 5011 married women aged 18–40 years who were planning to become pregnant in the next year were recruited from 20 communes in Thai Nguyen, a northern mountainous province of Vietnam.

All eligible women were randomly assigned to one of three pre-pregnancy groups for weekly supplementation of: 1) folic acid (FA, 2800 μg – control), 2) iron and folic acid (IFA, 2800 μg FA+ 60 mg iron) or 3) multiple micronutrients (MM, 15 micronutrients including 2800 μg FA+ 60 mg iron). The micronutrient composition of the supplements is shown in Additional file 1. Village health workers (VHWs) visited women every 2 weeks to deliver the supplements. They directly observed the consumption of one supplement during the home visit, and recorded the number of supplements consumed and any symptoms or side effects women may have since the last visit. They also verified the supplement packets stored at women’s homes and counted the number of remaining capsules. Compliance was calculated as the percentage of number of supplements consumed over the total number of supplements delivered. VHWs also asked women whether they had their menses since the last visit. Women who reported their last menstrual period to be >5 weeks were invited to the Commune Health Center (CHC) for a pregnancy test. Once women were confirmed as pregnant, they stopped weekly supplementation and started receiving supplements containing 60 mg of iron and 400 μg of FA that were consumed daily through delivery as recommended by the World Health Organization [17]. These women were scheduled for prenatal and postpartum visits at the CHC. All investigators, study personnel and participants were blinded to group assignment.

### Sample size estimations

The sample size calculations were based on the primary study outcomes (birth weight). Using a one tailed test of comparing means between any two groups, we had estimated that a minimum sample of 550 infants per group (1650 total) was needed to detect an effect size of 0.15 SD units or greater (60 g for birth weight) at a level of significance of 0.05 and 80% power. [18]. This sample size has at least 80% power to detect the difference of 0.5 scores in depressive symptoms during pregnancy or postpartum.

### Randomization and blinding

A list of all WRA was obtained from the CHC for each commune. VHWs visited the homes of all married non-pregnant women and invited those who were planning to become pregnant within the next year to participate in the screening. Women who were pregnant, had consumed IFA or MM supplements in the previous 2 months, were severely anemic (Hb < 7 g/L), or had a history of high risk pregnancy or chronic hematological diseases were not included in the study. Women with severe anemia were referred to the local health clinic for treatment and those with moderate and mild anemia were counselled about risks associated with pregnancy and anemia. Upon confirmation of eligibility, lists of the ID numbers of eligible women were used to generate a randomization code to assign women to one of the supplement groups.

All investigators, study personnel and participants were blinded to group assignment. All supplements were in capsule form, identical in appearance and taste, and coded with lot numbers at the factory that correspond to one of the three treatment arms. The code allocations were stored by two individuals not involved in the study at TUMP and Emory University and were opened only after completion of the trial.

### Measurements

#### Assessment of symptoms of depression:

We assessed maternal depressive symptoms using the Center for Epidemiologic Studies Depression Scale (CES-D) [19] before conception (baseline), and the Edinburgh Postnatal Depression Scale (EPDS) during pregnancy and at 3 months postpartum. During pregnancy, EPDS was administered at each prenatal visit up to three times: the first time was immediately after detecting pregnancy, normally during the first trimester, and the following two measures took place in the second and third trimesters of pregnancy.

The CES-D scale includes a 20-item checklist that measures symptoms of depression experienced over the past week, including depressed mood, guilt, worthlessness, helplessness, hopelessness, psychomotor retardation, loss of appetite, and sleep disturbance. Each question is scored on a scale of 0–3, yielding a range of possible scores from 0 to 60, with higher scores indicating increased severity of depressive symptoms. Several studies have adapted this instrument for use with a variety of populations [20, 21], including the Vietnamese population [22] and have confirmed its high validity and reliability. Baseline CES-D scores were categorized into tertiles (high, medium, and low) because very few women had values >16 which has been used in previous studies as a cut-off to be indicative of depression [19]. This allowed us to identify women with higher levels of symptoms relative to the other women within this sample of women.

The EPDS is a 10-item self-rating questionnaire that addresses symptoms of depression present in the last 7 days [23]. Each question has four alternative answers scoring 0 to 3, for a maximum score of 30; higher scores denote greater levels of depressive symptomatology. The EPDS has been used extensively to assess maternal mental health both during pregnancy and postpartum and has been used previously in Vietnam [24]. Symptoms of depression during pregnancy and postpartum were reported both as continuous outcomes (mean ± SD) of EPDS and as the proportion of women with high risk of maternal depressive symptoms (MDS), defined as EPDS score ≥ 4. This cut-off was selected based on previous work in Vietnam, where it was validated as a screening tool (with sensitivity 70%; specificity 73%) [25] and was predictive of infant motor development [24].

Both scales were translated into Vietnamese and checked for accuracy through back translation and pilot testing. The adaptation and administration of both these instruments were reviewed by the study psychologist (ADG) who has experience in using measures of depression in other studies [26, 27]. Both instruments were interviewer-administered. Interviewers were trained on the protection of interviewee privacy and data confidentiality. All interviews were conducted in private.

#### Biochemical indicators of micronutrient status:

A finger-prick capillary blood sample was obtained to measure hemoglobin (Hb) concentrations using a B-Hemoglobin analyzer (Hemocue). Venous blood samples were collected after overnight fasting at baseline, first prenatal visit and 3 months post-partum to measure iron store (plasma ferritin) using the sandwich ELISA technique [28].

#### Other characteristics

Participants were interviewed at baseline (before conception) to gather information on their demographic characteristics, level of education, current marital status, reproductive history, and health status [16]. Socio-economic status (SES) was created by principal components analysis [29] using a set of questions related to ownership of property and land, household assets, housing conditions, and access to utilities. Dietary intakes were collected at baseline using a semi-quantitative food frequency questionnaire developed by the Vietnam National Institute of Nutrition. This food frequency questionnaire was validated with Vietnamese adults [30]. Body weight and height were measured to the nearest 0.1 kg or 0.1 cm, respectively, and body mass index (BMI) was calculated as weight in kg over height in squared meters. All interviewers were well-trained in interviewing techniques, ethical issues and administration of the questionnaires.

### Statistical analysis

We first analyzed the outcomes using an intention-to-treat approach, followed by per protocol comparisons that restricted the analytic sample to women who had received at least 26 weeks of the preconceptional intervention. Proportions, means, and SDs were presented for selected baseline variables and compared between groups using ANOVA for continuous variables and chi-square tests for proportions. We examined the distributions of the CES-D and EPDS scores and compared mean values using ANOVA. We also conducted stratified analyses by selected baseline characteristics that had been chosen a-priori (preconception depressive symptoms, anemia, and maternal undernutrition- BMI < 18.5 kg/m^2^) using generalized linear models with Hosmer-Lemeshow goodness of fit test. Data analysis was performed using STATA 13.

## Results

**Fig. 1:**
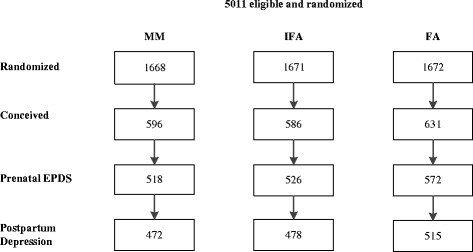
Flow diagram of participant progress throughout the study. EPDS - Edinburgh Postnatal Depression Scale, FA - folic acid, IFA - iron and folic acid, MM - Multiple micronutrient

Among 5011 women randomized for inclusion into the study, 1813 women became pregnant and contributed 1639 live births. Intent to treat analyses were conducted for 1616 women who had at least one depression measurement during pregnancy and 1465 women with data for postpartum depression (Fig. 1). Selected baseline characteristics including age, education, occupation, SES, dietary intakes, anthropometric measurements, and biochemical indicators were not significantly different across the three treatment groups (Table 1). Women were on average 26 years old and over 90% already had one child. Nearly a third were underweight (BMI <18.5 kg/m^2^), 20% had anemia, and 14% had depleted iron stores at baseline. Comparison of baseline characteristics also revealed no differences between women who had EPDS data available versus those who did not (results not shown). As reported previously, compliance was over 90% for both preconception and prenatal supplements and did not differ by treatment group [31]. The average duration of consumption of supplements before conception was 56 ± 28 weeks (ranged: 2–107 weeks). There were also no differences among the three groups in measures of weight and anemia at the first and second prenatal visit (result not shown).

**Table 1:** Selected maternal characteristics at baseline by intervention group

Variable^b^	MM (*n* = 518)^a^	IFA (*n* = 526)^a^	FA (*n* = 572)^a^	*P*-Value^c^
Age at randomization, y	26.1 ± 4.6	25.9 ± 4.2	25.8 ± 4.2	0.49
Age at first married, y	21.7 ± 3.2	21.8 ± 3.3	21.9 ± 3.3	0.70
Ethnic minority, %	52.8	48.9	48.4	0.29
Education Level, %				
Primary school	9.5	7.2	7.5	0.34
Secondary school	52.7	53.0	55.4	
High school	27.4	26.6	23.4	
College or higher	10.4	13.1	13.6	
Work as farmers, %	82.0	78.1	78.0	0.18
SES index	−0.01 ± 0.93	0.06 ± 0.95	0.06 ± 0.95	0.48
Number of children, %				
0	5.1	5.9	5.5	0.84
1	93.3	92.7	93.7	
≥ 2	1.6	1.3	0.8	
Anthropometric measurements				
Height, cm	152.7 ± 4.9	152.6 ± 5.0	152.8 ± 5.2	0.75
Weight, kg	46.1 ± 5.7	45.4 ± 5.1	45.8 ± 5.5	0.13
BMI, kg/m^2^	19.8 ± 2.1	19.5 ± 1.9	19.6 ± 2.1	0.10
Underweight (BMI < 18.5), %	29.0	31.5	32.2	0.50
Dietary intake /d				
Total energy, kcal	2240 ± 775	2249 ± 754	2183 ± 711	0.29
Iron, mg	17.3 ± 8.1	17.7 ± 7.5	17.0 ± 8.1	0.35
Zinc, mg	10.6 ± 3.6	10.6 ± 3.5	10.3 ± 3.6	0.19
Vitamin C, mg	235 ± 158	244 ± 145	245 ± 199	0.53
Folate, μg	344 ± 210	355 ± 190	343 ± 193	0.53
Vitamin B12, μg	2.1 ± 2.3	2.0 ± 1.7	1.9 ± 1.8	0.18
Vitamin A, μg	440.2 ± 496.9	424.3 ± 360.1	414.8 ± 379.8	0.60
Hematological indicators				
Hemoglobin, g/dL	12.9 ± 1.4	12.9 ± 1.4	13.0 ± 1.3	0.19
Anemia (Hb <12 g/dL), %	21.6	20.1	17.9	0.31
Ferritin, μg/L (geometric mean)^d^	66.31	64.07	66.89	0.74
Insufficient iron stores (ferritin <30 μg/L), %	13.5	14.6	11.1	0.20
Compliance to preconception supplements^e^	95.5 ± 9.7	96.1 ± 8.7	95.7 ± 9.4	0.19
Compliance for prenatal supplements^e^	97.1 ± 9.1	97.7 ± 6.9	97.7 ± 7.1	0.27

^a^Sample sizes for each variable vary slightly due to item-specific missing data

^b^Values are mean ± SD or percentages unless otherwise noted

^c^ANOVA test for comparison of means and goodness of fit test for comparison of proportions

^d^Ferritin values were adjusted for inflammation indicators

^e^Compliance was calculated as the percentage of number of supplements consumed over the total number of supplements delivered

*AGP* alpha-1-acid glycoprotein, *CRP* C- reactive protein. *BMI* Body Mass Index, *FA* Folic acid, *IFA* Iron and folic acid, *MM* Multiple micronutrient, *SES* Social Economic Status, *RBP* Retinol Binding Protein

Preconceptional (baseline) mean CES-D score was 3.48 ± 4.71 and was similar across the three groups. Mean EPDS scores during pregnancy was 1.5 ± 2.7, 1.1 ± 2.4, 0.7 ± 2.0 at first, second and third trimesters, respectively, and 0.6 ± 1.8 during the early postpartum period. The proportion of women ever experiencing elevated MDS during pregnancy was 11.3%, 8.1% and 4.9% at first, second and third trimesters, respectively, and 3.6% during the early postpartum period. We found no significant differences by treatment group for mean EPDS scores or prevalence of elevated MDS during pregnancy and 3-months postpartum (Table 2). The per-protocol analysis also showed no differences in elevated MDS by treatment group in the subgroup of women who received the supplement for at least 26 weeks before conception (results not shown).

**Table 2:** Mean depression scores and proportion of women at risk of elevated depressive symptoms from preconception to postpartum by intervention group

Variable^b^	MM (*n* = 518)^a^	IFA (*n* = 526)^a^	FA (*n* = 572)^a^	*P*-Value^c^
Mean depression score				
Preconception CESD	3.42 ± 4.58	3.33 ± 4.13	3.66 ± 5.30	0.48
Prenatal EPDS (first trimester)	1.43 ± 2.57	1.32 ± 2.43	1.62 ± 3.03	0.15
Prenatal EPDS (second trimester)	1.03 ± 2.30	1.15 ± 2.38	1.18 ± 2.46	0.69
Prenatal EPDS (third trimester)	0.71 ± 1.94	0.61 ± 1.85	0.87 ± 2.16	0.20
Postpartum EPDS (3 mo)	0.65 ± 1.91	0.56 ± 1.57	0.59 ± 1.98	0.72
Proportion who reported elevated MDS during pregnancy				
Prenatal (first trimester)	11.00	9.32	13.29	0.11
Prenatal (second trimester)	7.42	8.94	8.04	0.68
Prenatal (third trimester)	6.08	3.11	5.29	0.16
Postpartum (3 mo)	4.45	3.32	0.31	0.49

^a^Sample sizes for each variable vary slightly due to item-specific missing data.

^b^Values are mean ± SD or percentages unless otherwise noted

^c^ANOVA test for comparison of means and goodness of fit test for comparison of proportions

*CESD* Center for Epidemiologic Studies Depression Scale, *EPDS* Edinburgh Postnatal Depression Scale, *FA* Folic acid, *IFA* Iron and folic acid, *MDS* Maternal depressive symptoms, *MM* Multiple micronutrient

**Fig. 2:**
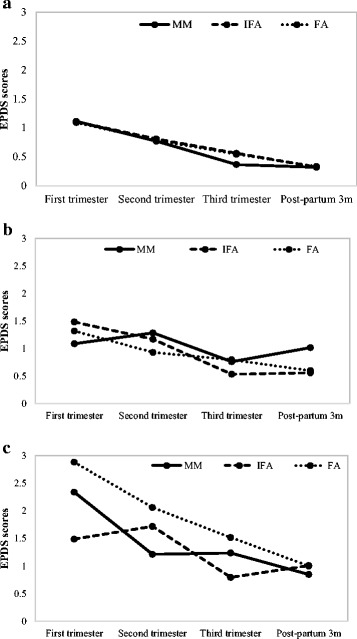
Mean EPDS score during pregnancy and postpartum, by treatment group and baseline level of depressive symptoms^1,2^.**a** Low tertile CESD score (*n* = 725), (**b**) Middle tertile CESD score (*n* = 468), (**c**) High tertile CESD score (*n* = 423). ^1^ *P* = 0.001 and 0.047 for interaction between treatment group and high tertile level of depressive symptoms for mean EPDS score at first and second trimester, respectively ^2^. Baseline CESD score: low tertile: score 0–1, middle tertile: score 2–4. High tertile: score 5–46.*CESD* Center for Epidemiologic Studies Depression Scale, *EPDS* Edinburgh Postnatal Depression

The results of stratified analyses showed differential impact of the intervention on EPDS scores during pregnancy by baseline depression status. Among women in the highest tertile for CES-D scores at baseline, mean EPDS scores were significantly lower in the first (MM:2.34, IFA:1.45, FA: 2.88) and second trimester (MM:1.21, IFA: 1.27, FA:2.06) of pregnancy in the MM and IFA groups compared to FA only (*P < 0.05*) (Figure 2). In contrast, there was no difference by treatment group on the risk of MDS during the postpartum period. There were also no differences by treatment group by maternal underweight or anemia (results not shown) for mean EPDS or risk of elevated MDS during pregnancy or the post-partum period.

## Discussion

In this large randomized controlled trial, we found no main effect of weekly preconceptional supplementation with MM or IFA compared to FA on prenatal and postpartum depressive symptoms among women of reproductive age from northern Vietnam. These findings were the same for the per-protocol analysis, which included women who consumed the supplements for at least 6 months prior to conception. Iron deficiency and anemia have been associated with fatigue [32, 33] and increased post-partum depressive symptoms [12], and it is possible that prenatal IFA supplements, which were received by all women after conception, may have benefited all women. The lack of differences by intervention groups in weight or anemia both during pregnancy and early post-partum also suggest possible attenuation of the preconception intervention. We have also shown that iron status was better during early pregnancy in the MM and IFA group compared to FA only, but there were no differences in maternal iron status at 3 months post-partum or in cord blood ferritin [34]. Finally, the lack of differences may be also due to the fact that all groups received FA which has been shown to reduce depressive symptoms [35].

However, we found evidence of selective effects among women at risk for depression before conception: among women in the highest tertile of CES-D scores at baseline, women who received MM or IFA before conception had lower mean EPDS scores in the first and second trimesters of pregnancy compared to those who received only FA. These differences were statistically significant (*p* < 0.05). However, the difference is small and may not be clinically significant. Various micronutrients, such as vitamin D, selenium, zinc, vitamin B12, folates and other B vitamins, have been suggested as important for the prevention of maternal depression [36]. Since iron and folic acid were common to both MM and IFA, and all groups received FA, we assume that iron was the key element explaining the results, but the mechanisms are unclear since we did not find differential effects of the intervention by baseline iron status or anemia, and the effect of MM and IFA supplements among those at-risk for depression at baseline was not observed during the postpartum period. While previous studies have shown an association between iron status and maternal depression postpartum [11, 12], a recent study in China reported only small correlations between iron status throughout pregnancy and postpartum depressive symptoms measured using EPDS [37].

Prevalence of elevated MDS decreased significantly as pregnancy progressed, and was even lower during the early postpartum period. This pattern is consistent with the other study in Vietnam using a similar cut-off; however, the prevalence of high depressive symptoms during pregnancy was lower compared to this previous study [24] (~11% vs. 40% in early and 5% vs. 28% in late pregnancy), and much lower (~ 3%) when using a standard cut-off (EPDS > 10) than what has been reported in low and middle income countries (16%) [38]. In addition, in this cohort, the prevalence of high depressive symptoms was very low in the post-partum period. It is estimated that post-partum depression ranges between 10 and 15% worldwide [39]. However, country-specific variation has been reported, with some countries such as Singapore, Malta, Malaysia, Austria and Denmark reporting almost no postpartum-depression and others including Brazil, Costa Rica, Taiwan and Korea reporting a high prevalence of up to 60% [39]. One study from Vietnam reported a prevalence of 33% for depression at 6 weeks postpartum in an urban population/hospital setting from Ho-Chi-Min City [40]. This contrasts with the extremely low prevalence of ~1% in our cohort at 3 months post-partum if using the standard cut-off (EPDS > 10). The differences may be due in part to our study selection criteria and timing of measurement; we included only married women who clearly expressed their intention to become pregnant, and women with planned pregnancies have been shown to have lower rates of postpartum depression compared to those with unplanned pregnancies [41]. Additionally, our study was conducted in a more rural population and reporting of depressive symptoms might be different between urban and rural women.

Strengths of this study include the large sample size, the extensive follow-up from preconception through pregnancy and delivery, the randomized controlled trial design, the double blinding to the intervention of participants and the research team, and the extensive data collection on socio-demographic, physiological, and mental health variables at different time points. The field personnel were extensively trained and procedures were standardized to assure high quality data-collection. To our knowledge, this is the first study to assess the impact of preconceptional micronutrient supplementation on maternal depressive symptoms during pregnancy and after delivery. Conversely, limitations of this study include the lack of biomarkers of micronutrient status except for iron. Although we have information on underweight and dietary quality, these may be imperfect proxies to account for possible differences. We also did not measure perceived level of social and/or emotional support by these women which may be associated with depressive symptoms [42]. Our population was unique in the low-prevalence of iron-deficiency and postpartum depression; thus our results might not be generalizable to other populations where these two factors are of greater concern. As mentioned before, women enrolled in this study were highly motivated to get pregnant, which might also affect the generalizability of our findings to other populations at higher risk of post-partum depression.

## Conclusion

Weekly preconceptional micronutrient supplements containing iron did not improve depression measures relative to folic acid alone among all women, but may have benefitted women who were at risk for depression. However, the mechanisms are unclear, and they may have been due to chance especially since we did not see any interactions by baseline markers of iron status. Future studies are needed to evaluate the benefits of providing preconceptional multiple micronutrient supplements in other populations with greater prevalence of iron-deficiency and/or maternal depression and the optimal package of services that is needed to promote the health and wellbeing of women of reproductive age.

## Additional files

Additional file 1: The composition of iron folate and multiple micronutrient supplements. (DOCX 14 kb)

Additional file 2: Dataset. (XLSX 1042 kb)

## Abbreviations

BMI: Body Mass Index
CES-D: Center for Epidemiologic Studies Depression Scale
CHC: Commune health center
EPDS: Edinburgh Postnatal Depression Scale
FA: folic acid
IFA: iron and folic acid
MDS: maternal depressive symptoms
MM: multiple micronutrient
RCT: randomized controlled trial
VHW: Village health worker
