# Supplementary material for: Impact of preconceptional micronutrient supplementation on maternal mental health during pregnancy and postpartum: results from a randomized controlled trial in Vietnam
Source: BMC Womens Health. 2017 Jun 17;17:44. doi: 10.1186/s12905-017-0401-3 (PMC5473979; doi:10.1186/s12905-017-0401-3)
Supplement: Supplementary file 1 — The composition of iron folate and multiple micronutrient supplements. (DOCX 14 kb) [file 12905_2017_401_MOESM1_ESM.docx]

**Additional file 1: Table S1 The composition of iron folate and multiple micronutrient supplements**

| **Ingredient** | **Pre-pregnancy (weekly)** | | | **RDA for non-pregnant women** |
| --- | --- | --- | --- | --- |
|  | **FA** | **IFA** | **MM** |  |
| Vitamin A (μg) |  |  | 800 | 700 |
| Vitamin D (IU) |  |  | 600 | 15 |
| Vitamin E (mg) |  |  | 10 | 15 |
| Vitamin C (mg) |  |  | 70 | 75 |
| Thiamine (mg) |  |  | 1.4 | 1.1 |
| Riboflavin (mg) |  |  | 1.4 | 1.1 |
| Niacin (mg) |  |  | 18 | 14 |
| Vitamin B_6_ (mg) |  |  | 1.9 | 1.3 |
| Vitamin B_12_ (μg) |  |  | 2.6 | 2.4 |
| Folic acid (μg) | 2800 | 2800 | 2800 | 400 |
| Iron (ferrous sulfate) (mg) |  | 60 | 60 | 18 |
| Zinc (sulfate) (mg) |  |  | 15 | 8 |
| Copper |  |  | 2 (mg) | 900 (μg/d) |
| Selenium (μg) |  |  | 65 | 55 |
| Iodine (μg) |  |  | 150 | 150 |

FA - folic acid, IFA - iron and folic acid, MM - Multiple micronutrient, RDA - Recommended dietary allowance
